# Functionalized Anatomical Models for Computational Life Sciences

**DOI:** 10.3389/fphys.2018.01594

**Published:** 2018-11-16

**Authors:** Esra Neufeld, Bryn Lloyd, Beatrice Schneider, Wolfgang Kainz, Niels Kuster

**Affiliations:** ^1^IT'IS Foundation for Research on Information Technologies in Society, Zurich, Switzerland; ^2^Ärzteteam 51, Arbeitsmedizin Brugg, Brugg, Switzerland; ^3^Division of Biomedical Physics, OSEL, CDRH, Food and Drug Administration, Silver Spring, MD, United States; ^4^Swiss Federal Institute of Technology (ETHZ), Zurich, Switzerland

**Keywords:** computational life sciences, computational phantom, anatomical model, functionalization, simulation, modeling

## Abstract

The advent of detailed computational anatomical models has opened new avenues for computational life sciences (CLS). To date, static models representing the anatomical environment have been used in many applications but are insufficient when the dynamics of the body prevents separation of anatomical geometrical variability from physics and physiology. Obvious examples include the assessment of thermal risks in magnetic resonance imaging and planning for radiofrequency and acoustic cancer treatment, where posture and physiology-related changes in shape (e.g., breathing) or tissue behavior (e.g., thermoregulation) affect the impact. Advanced functionalized anatomical models can overcome these limitations and dramatically broaden the applicability of CLS in basic research, the development of novel devices/therapies, and the assessment of their safety and efficacy. Various forms of functionalization are discussed in this paper: (i) shape parametrization (e.g., heartbeat, population variability), (ii) physical property distributions (e.g., image-based inhomogeneity), (iii) physiological dynamics (e.g., tissue and organ behavior), and (iv) integration of simulation/measurement data (e.g., exposure conditions, “validation evidence” supporting model tuning and validation). Although current model functionalization may only represent a small part of the physiology, it already facilitates the next level of realism by (i) driving consistency among anatomy and different functionalization layers and highlighting dependencies, (ii) enabling third-party use of validated functionalization layers as established simulation tools, and (iii) therefore facilitating their application as building blocks in network or multi-scale computational models. Integration in functionalized anatomical models thus leverages and potentiates the value of sub-models and simulation/measurement data toward ever-increasing simulation realism. In our o^2^S^2^PARC platform, we propose to expand the concept of functionalized anatomical models to establish an integration and sharing service for heterogeneous computational models, ranging from the molecular to the organ level. The objective of o^2^S^2^PARC is to integrate all models developed within the National Institutes of Health SPARC initiative in a unified anatomical and computational environment, to study the role of the peripheral nervous system in controlling organ physiology. The functionalization concept, as outlined for the o^2^S^2^PARC platform, could form the basis for many other application areas of CLS. The relationship to other ongoing initiatives, such as the Physiome Project, is also presented.

## 1. Introduction

Human and animal anatomical models, highly detailed geometric representations of the distribution of different tissues in the body, have established themselves as crucial components not only for the dosimetric assessment of ionizing/non-ionizing radiation exposure, but also, more recently, in computational life sciences (CLS)—e.g., to gain mechanistic understanding, to develop novel therapeutic approaches and medical devices, or to demonstrate the safety of implants in magnetic resonance environments. The greater vision is that they can be routinely applied for treatment personalization and optimization, and to complement or replace classical animal or human clinical trials by *in silico* clinical trials for the assessment of safety and/or efficacy. The first ever created, highly detailed anatomical models for dosimetric simulation purposes were based on the cryosection data from the Visible Human project (Spitzer et al., 1996). Thanks to advances in imaging technologies, such models are now mainly based on non-invasive imaging, thus enabling the creation of representations of broader sections of the population and of personalized anatomical models. One example is our Virtual Population (ViP), a set of highly detailed anatomical models (Christ et al., 2009; Gosselin et al., 2014) generated from magnetic resonance imaging (MRI) data of male and female adults and children across a wide range of ages, including models of an elderly and an obese male. To date, the ViP has been used in over 1000 publications and over 200 regulatory submissions worldwide. Another widely employed set of models are the XCAT phantoms (Segars et al., 2018). These are NURBS-based models (whereas the ViP employs triangle meshes) that are particularly popular in ionizing radiation dosimetry research.

The rise of computational modeling, involving complex anatomical models, has been fueled by progress in simulation methodology, available computational power, and the existence of a larger number of suitable anatomical models (Xu, 2014). The latest generation of anatomical models, such as VIP 3.0, provides the necessary detailed 3D tissue (and tissue properties) distributions to determine whole-body and local physical exposure and interactions. However, the anatomical representation of the body cannot be entirely separated from the modeling of living tissue and (organ) physiology within it. Physical exposure from medical devices/therapies is the source of tissue interactions that affect (with therapeutic benefit or adversely) physiology, while physiology affects exposure. For example, dielectric tissue properties affect electromagnetic (EM) exposure in neurostimulation applications, resulting in modulation of electrophysiological function. In turn, physiological activity, such as breathing, can result in changes to the anatomical geometry, which then affects the medical device function (e.g., a radiotherapy treatment). Local energy absorption results in a thermoregulatory response that determines the final tissue temperature distribution. Hence, there is the need to bring anatomical and physiology representation closer together. Support in this endeavor is offered by recent rapid advances in medical imaging, which provide access to detailed information about the anatomy, physiological dynamics, and tissue properties and behavior, as well as by an increased understanding of the physiology and its computational representation.

In this paper, we present our vision of functionalized anatomical models as a paradigm for CLS (a more limited neuro-functionalized anatomical model concept has been formulated in Neufeld et al., 2016a). The goal is to discuss different forms that model functionalization can take, as well as how they can converge in a comprehensively functionalized general model, and to illustrate them with practical examples drawn from our efforts toward generating such models. Furthermore, we introduce and argue the concept of using functionalized anatomical models as integration centers for heterogeneous models at various scales and data in larger CLS initiatives, using the o^2^S^2^PARC platform as example (section 2.3).

It should be noted that there is a partial overlap between the functionalized anatomical model concept presented here and Physiome initiatives such as the International Union of Physiological Sciences (IUPS) and the Virtual Physiological Human (VPH) Physiome Projects (Bassingthwaighte, 2000; Hunter and Borg, 2003; Hunter and Viceconti, 2009). These initiatives aim at characterizing an individual's or species' physiological state (i.e., the physiome) through “databasing” and integrated modeling (Bassingthwaighte, 2000). While part of the functionalized anatomical model concept can be seen as a realization of the physiome vision in that it involves integration of physiological data and computational models, the functionalization concept is distinct in several ways as it (i) finds its primary application in the context of physical interactions between the environment or medical devices with the human body and physiology, (ii) focuses on the strong link between (whole body) anatomy and physiological dynamics, and (iii) pursues a more heterogeneous and flexible integration – even though this is typically associated with looser integration and weaker coupling – compared to the strict multi-scale model integration promoted in the Physiome Project (through common markup language descriptions and imposition of conservation laws across model components; Hunter, 2016). The differences between the Physiome initiatives and our vision for functionalized anatomical models (e.g., the advantages and disadvantages of heterogeneous coupling) are discussed in more detail in section 3.1. In addition, the relationship to precision medicine visions, such as the digital patient “Avatar,” is discussed in view of the potential and benefit of creating personalized functionalized anatomical models.

The employed technologies presented here, such as performing simulations with the help of anatomical models and co-registered data or computational models, are *per se* not novel. However, the goal of this paper is to argue (i) for the systematic creation of functionalized anatomical models that are based on sufficiently standardized building blocks to facilitate the coupling and linking of a range of computational/anatomical models and simulation/measurement data, as well as (ii) the concept that they can serve as integration services for unified computational frameworks or platforms.

## 2. Methods and results

This section consists of two parts: First (section 2.2), different forms of functionalization are described and illustrated with concrete application examples from the literature. These examples are mostly taken from our own work with the anatomical models summarized in section 2.1. Based on the lessons learned about the value of integrating functionalization layers within anatomical models and on the related concepts that have been established, we put functionalized anatomical models at the center of the o^2^S^2^PARC platform, a CLS platform initially developed in the context of investigating peripheral nervous system (PNS) control of organ physiology, as described in section 2.3.

### 2.1. Anatomical models

**Figure 1:**
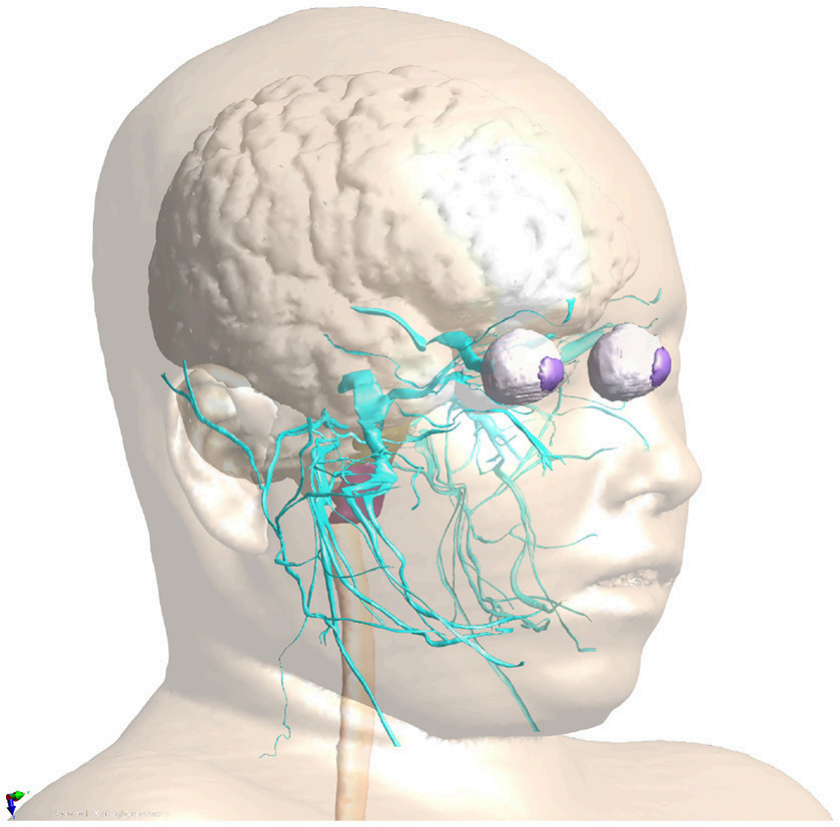
PNS functionalization of NEUROMAN: The cranial nerve traced for the basic version of NEUROMAN are shown. They have been obtained by segmentation of color cryosection images. These trajectories are complemented with dynamic electrophysiology models. Within the SPARC initiative, additional nerve trajectories and electrophysiology models elaborated by SPARC teams will be successively integrated.

Most of the examples discussed in this paper involve one of the following anatomical models:

- Virtual Population (Christ et al., 2009; Gosselin et al., 2014): The ViP includes a range of 11 detailed models (s. section 1). It is unique in that it provides wide population coverage based on painstakingly segmented image data from healthy volunteers, rather than relying solely on morphing of a single underlying model. The image data has been segmented using a range of automatic and manual methods. Subsequently, triangular surfaces have been extracted, smoothed, and simplified, with methods that ascertain conformality of adjoining regions (no gaps or overlaps) and absence of (self-)intersections.
- MIDA (Iacono et al., 2015): MIDA is a detailed anatomical human head and neck model with over 160 distinguished structures. The full version includes a detailed segmentation of thalamic and subthalamic nuclei, which had to be removed from the freely available version for IP reasons. The distinguishing feature of the MIDA model (in addition to the high resolution and detailedness) is that it is based on a broad, multi-modal set of MRI images acquired in a single session from one volunteer. That set includes differently weighted (T1, T2, high nerve contrast) structural images, diffusion tensor imaging (DTI), as well as phase-contrast and time-of-flight angiography. The multimodal image data facilitates detailed segmentation (e.g., the two blood flow imaging modalities facilitated separation of arteries and veins), but also provides valuable information for functionalization (s. section 2.2.2). The anatomical model generation approach is similar to that used for the ViP models.
- NEUROMAN (Lloyd et al., 2018): NEUROMAN (Figure 1) are two evolving anatomical models based on high resolution cryosection image data from a Korean female and male (Park et al., 2006; Yeom et al., 2014). In addition to the unique quality of the underlying color-image data and the large number of identified tissues, the distinguishing feature of NEUROMAN is the effort toward adding an extensive tracing of the PNS to the model. The aim is to functionalize the PNS trajectories with electrophysiological neuron models, a concept developed in recent pilot studies (Neufeld et al., 2016b; Cassara et al., 2017b). Foreseen applications are manifold and include, e.g., risk assessment of low-frequency exposures from high-power wireless power transfer systems (Reilly and Hirata, 2016) or the development of safe MRI sequences with improved contrast and resolution.

### 2.2. Functionalization forms

Functionalization describes the integration of additional model layers into the static, geometrical anatomical models. There are different forms of functionalization—depending on the kind of dynamics, computational model, or data to be integrated—which are discussed below.

#### 2.2.1. Geometry functionalization

The initial anatomical model is a static geometric representation of the different tissues, organs, and regions. There can be different reasons, why the geometry should be rendered dynamic or parameterized:

- The anatomy of the patient/subject undergoes rapid changes on the time-scale of treatments (e.g., related to breathing, heartbeat, bowel movement, organ sliding after changing from standing to resting position). The deformed anatomy can affect exposure, e.g., during proton-beam therapy. Similar considerations apply to posture changes.
- The anatomy of the patient/subject undergoes slow changes on the time-scale of treatments (e.g., due to tumor shrinkage during radiotherapy).
- The population coverage needs to be increased (e.g., in the context of *in silico* clinical trials) to better account for anatomical variability. This can be achieved by parameterizing anatomical models with gross anatomical measures such as height or body-mass-index (BMI).
- Personalized models need to be created, e.g., by registering an existing anatomical model to (image) data of a person.

**Figure 2:**
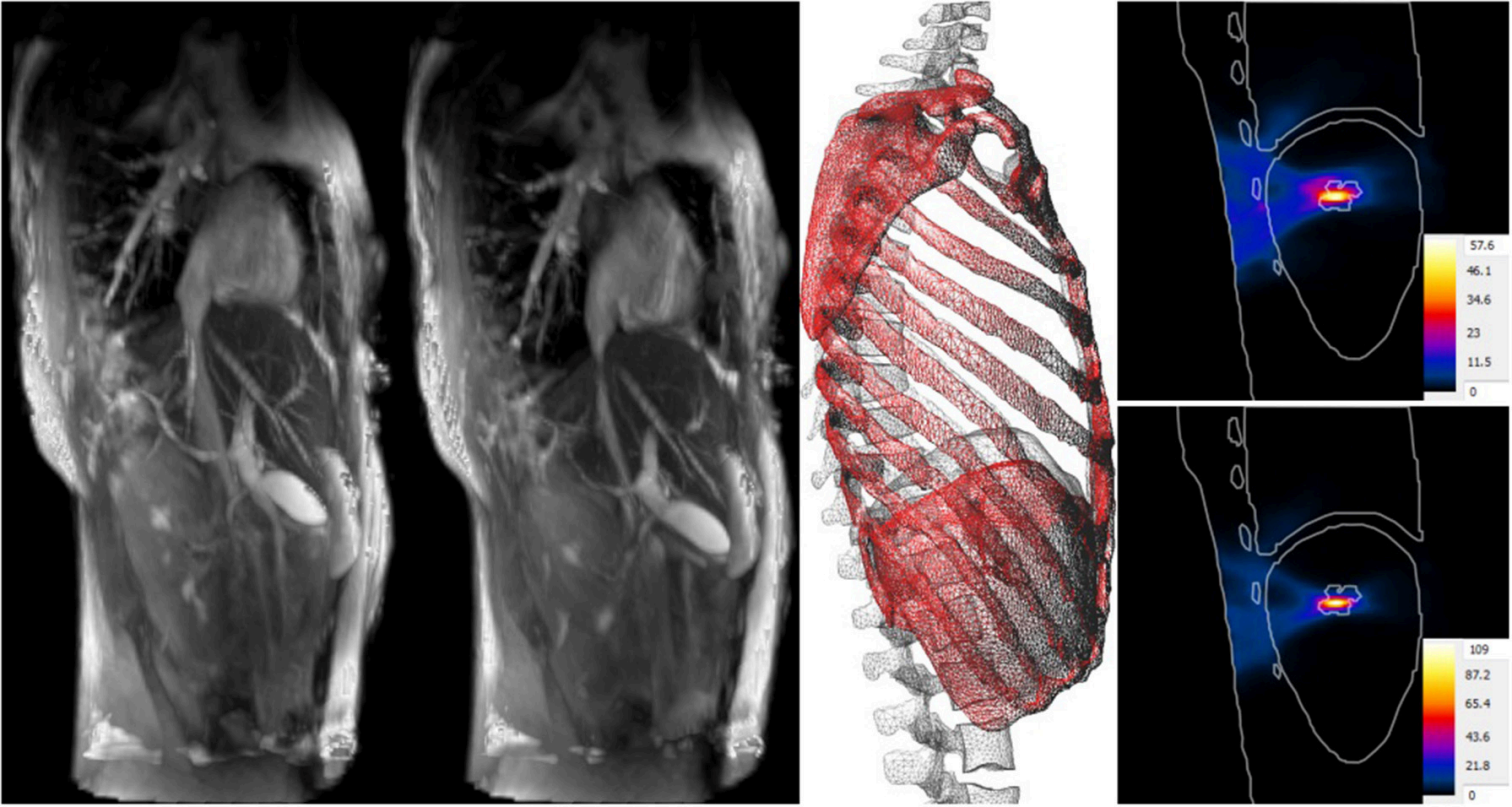
Impact of breathing motion on focused ultrasound liver ablation. 4D MRI data (**A** at maximal exhalation, **B** at maximal inhalation) is used to create deformation fields that are registered to the anatomical model, thus functionalizing it with breathing motion **(C)**. Compared to the temperature increase when neglecting motion **(D)**, tracking compensation **(E)** can double the theoretical temperature increase, result in better focusing, and reduce collateral damage.

The following techniques were developed to advance the ViP models beyond static geometries:

- Image registration: Registration of the anatomical model to image data can be used to personalize models, or to reflect motion and anatomical change. It is possible to either (i) register the original image data, used to generate the anatomical model, to specific image data (e.g., of that of a patient) or (ii) to directly register the anatomical model surfaces to the specific image data. When performing image-to-image registration, the imaging modalities do not necessarily need to agree. In some cases, it can be helpful to identify landmarks as registration constraints.
  For example, Kyriakou et al. (2014) functionalized a ViP model with a transient deformation field extracted from 4D MRI images to realistically model breathing motion and related organ deformation and displacement (Figure 2). The dynamic anatomical model was used to simulate the strong impact of breathing motion on focused ultrasound liver ablation therapies. It was concluded that breathing motion can lead to defocusing, collateral damaging near ribs, and the inability to reach therapeutic temperatures. However, considering breathing motion in the treatment planning and administration can mostly avoid these issues and can even be employed to achieve better coverage of large treatment areas.
- Biomechanical morphing: The muscle- or fat-content of anatomical models can be parameterized by performing biomechanical simulations in which shrinking or growing forces are associated with specific tissues, while other soft tissues are treated as passively deforming and bones as hard boundary conditions (Lloyd et al., 2016). A similar biomechanical approach can be applied to change the posture. A virtual skeleton consisting of “bones” and articulations is defined that can be posed, determining the displacement of some regions (mostly bones), while soft tissue again passively deforms, following the laws of biomechanics. Such an approach, while computationally demanding, produces more realistic posture changes, than, e.g., an influence region-based computation of deformation fields (Cherubini et al., 2009).
  Murbach et al. (2017b) investigated the dependency of local radiofrequency (RF) exposure during MRI on patient posture and obesity levels. Physics-based morphing was used to morph the obese male “Fats” ViP model to a similar BMI as the normal-weight “Duke” model. Combined morphing and posing allowed the testing of different hypotheses, identifying posture and BMI as key parameters for assessing RF exposure.
- Surface registration: Full-body anatomical models can be personalized to match gross anatomical characteristics of individuals by a three-step registration process (Lloyd et al., 2017; Alaia et al., 2018): first (i) the body surface of the template or reference model is registered to a target body shape (e.g., obtained from 3D laser scanner data), then (ii) various heuristics are used to reconstruct the estimated thickness distribution of the subcutaneous adipose tissue (SAT), and finally (iii) all internal tissues are registered based on a Poisson extension of the deformation field on the interior skin (i.e., the surface below the skin and SAT). The method is able to morph a complete model to a target body shape while ensuring anatomical correctness.
  Although organ sizes and shapes cannot be predicted solely based on the body shape, various tissues (e.g., muscle, SAT) and bones are reconstructed remarkably well, as evidenced in another MRI exposure safety study. Murbach et al. (2017a) used models with different body shapes, obtained by registering the ViP model “Ella” to female body shapes from the CAESAR project (Robinette et al., 1999). Based on the surface registration technique, various ViP models were registered to different body shapes. In order to analyze the predictive power and accuracy of the registered model, the Duke model was registered to Fats and the differences between the real Fats and the Duke-based Fats were analyzed (Alaia et al., 2017). Results revealed a remarkably good overlap of the overall body shape, bones and many other tissues. The internal structures of the template model agree with the internal structures of the target with relative surface area deviations of 1–15% between corresponding tissues and relative volume errors of 5–20%. A large difference was seen in the volume of the lungs, which is considerably below population average in Fats.
- Control-point (free-form) morphing: By overlaying a grid of control-points that can be freely shifted, a deformation field can be defined and applied to the model mesh. For example, a sequence of deformation fields mimicking breathing motion can be defined, or the shape of an organ parameterized (e.g., variable heart volume) (Neufeld et al., 2011).

With the exception of biomechanical morphing and posing, the described techniques must be considered as “tools” – providing them in combination with anatomical models does not yet constitute functionalization (there is no inherent connection between the transformed anatomical model and the transforming tool, as the use of the tool does not rely on the integration of information within the anatomical model). However, storing deformation fields (obtained through biomechanical morphing, image-registration, or principal component analysis of shape variability) along with the anatomical model is a form of functionalization that enhances the model's value in applications where motion or population coverage is relevant. The biomechanical morphing and the poser tool require model-specific information (e.g., “bone” and pivot system) and the combination of that information and the tool with the anatomical model is a form of functionalization.

Image- and surface-based registrations are important and powerful tools frequently employed in the context of functionalization. Beyond the use of registration tools to adapt general anatomical models to subject specific information, co-registration is crucial when merging information originating from different modalities into a synchronized functionalized model, as illustrated below. A wide range of techniques exist that can (co-)register image-data, segmentations, and surfaces to each other. Many of these have been applied in the context of the ViP and MIDA. However, discussing registration techniques further in more detail is outside the scope of this paper.

#### 2.2.2. Physics functionalization

Physical modeling is important, as physical interactions with tissues and/or physiology (e.g., from device-related exposure) is at the origin of most life sciences applications. Accurate determination of tissue exposure is therefore fundamental and requires proper specification of the physical characteristics and conditions present in the body. Anatomical model functionalization with regard to physical modeling can take various forms, which are described below.

##### 2.2.2.1. Tissue properties

Anatomical models provide discretization into distinct tissues that are associated with distinct physical properties. Linking an anatomical region to a set of tissue properties, such as density or dielectric properties, is a basic form of functionalization. Properties can be parameter-dependent, such as the frequency-dependence of acoustic properties, and that dependence can be derived from physical insights or from measurements. In some cases, it can be valuable to parameterize properties with regard to tissue composition – for example, age affects water content in many tissues and, therefore, also tissue properties. Through a tagging mechanism, the different regions in the ViP models are directly functionalized with material properties from the IT'IS tissue database (Hasgall et al., 2015). The database contains information about density, thermal properties, perfusion, dielectric properties (low-frequency conductivity and conductivity/permittivity dispersion relationships across a wide range of frequencies), frequency dependent acoustic properties, MRI relaxation times, element composition, and viscosity. It has been assembled through an extensive literature review and provides not only guidance on the most representative values for a wide range of tissues, but also on the variability of the reported values, which is an indicator of inter/intra-person variability and measurement uncertainty and thus required as part of modeling-uncertainty quantification.

**Figure 3:**
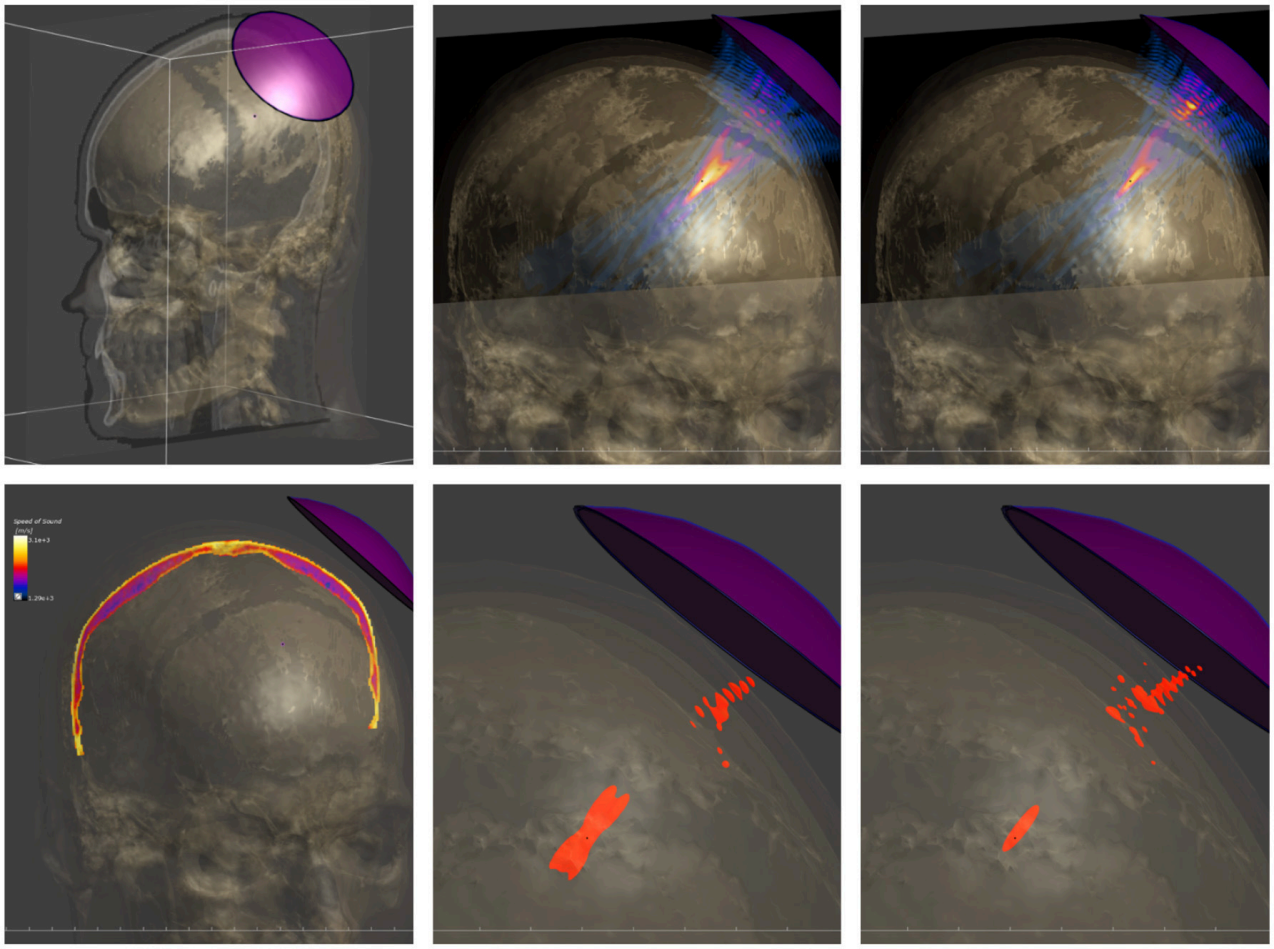
Modeling of transcranial focused ultrasound using a detailed head model functionalized with CT-based tissue property maps. **(Top)** Setup involving the head model and a single-element, curved acoustic transducer (left), simulated pressure distribution when neglecting skull heterogeneity (center) and when employing the functionalized head model (right). **(Bottom)** CT-based acoustic velocity map in skull (left) and half-peak pressure isosurfaces when neglecting skull heterogeneity (center) and when considering it (right). The latter model predicts lower pressure amplitudes, but a sharper focus with reduced interference side-lobes, as confirmed experimentally.

A form of tissue property functionalization that illustrates the functionalized anatomical model philosophy better, concerns tissue heterogeneity: The segmentation process involved in anatomical model creation reflects the simplifying approach of identifying regions that can be treated as somehow homogeneous. However, this can in some cases be overly simplistic. For example, bone has a highly heterogenous density. Imaging techniques can be used to collect information about such heterogeneity, such as MRI perfusion maps or computed tomography (CT) density maps. Co-registering such property maps along with anatomical models is a form of functionalization that makes this valuable information available for modeling purposes. The MIDA model provides functionalization with anisotropic (tensorial) low frequency conductivity maps, based on the co-registered DTI data. EM simulations of transcranial direct current stimulation (Iacono et al., 2015) demonstrate that consideration of the (inhomogeneous) anisotropy of neural tissue conductivity in a suitably functionalized head model results in a decrease of predicted field strength and penetration depth. The DTI information can also be used to trace neuron trajectories that can then be funtionalized with dynamic electrophysiological models. Similar to the use of DTI-derived electrical conductivity anisotropy maps in transcranial electric stimulation modeling is the use of anatomical head models functionalized with CT-based bone-property maps for the simulation of transcranial focused ultrasound (Figure 3). Simulations considering bone heterogeneity predict reduced sonication intensities, but improved focality (less side-foci), as confirmed by experimental measurements (Montanaro et al., 2018).

##### 2.2.2.2. Boundary conditions

Imaging-based information worth adding to anatomical models is not restricted to property distributions. Another case is the use of image data to impose boundary conditions on physics simulations. For example, various forms of imaging techniques are capable of determining (transient) blood flow rates in a vascular cross-section. Functionalization of anatomical models with such data adds additional realism when performing hemodynamics simulations within the anatomical vasculature model, as illustrated by Kyriakou et al. (2012). An anatomical model was functionalized with data from MRI flow velocimetry in 2D cross sections, which was then used as a transient boundary condition for blood flow simulations in the aorta and vena cava compartments. Subsequently, the ECG signal distortion due to the magneto-hemodynamic effect (present during high magnetic field exposure) was modeled using coupled EM simulations (Kainz et al., 2010), in order to assess its suitability as non-invasive biomarker for blood-flow features. The co-registered flow velocimetry data was used in the same study to validate the computational fluid-dynamics predictions, while co-registered surface potential measurement data served to validate the electric potential modeling predictions.

#### 2.2.3. Physiology functionalization

The next level of functionalization is concerned with adding tissue or organ physiology layers. The inclusion of physiology modulated tissue properties is conceptually situated between physics- and physiology-functionalization. For illustration purposes, we consider perfusion, which is an important parameter of the Bioheat Equation (Pennes, 1948) frequently employed to study heating or cooling of living tissue. Local vasodilation strongly depends on local temperature and, therefore, it can be necessary to associate tissues with tissue-specific, temperature-dependent perfusion models. While this is still readily viewed as a classic, non-constant material law, the situation changes, when the physiological complexity of thermoregulation is increasingly considered. In these cases, non-local variables (such as the median skin temperature or the temperature of the hypothalamus) come into play and depending on the situation, physiological models, e.g., for sweating or shivering, need to be included. Functionalizing anatomical models with physiological models, such as thermoregulation models, prepares them for applications beyond the classical exposure modeling.

Physiological functionalization is clearly not limited to tissue properties. For example, neuron trajectory/morphology models, complemented with models of their electrophysiology, can be included in anatomical models to study the impact of physical exposure within anatomical environments on physiological response. Going to the level of modeling physiology, instead of stopping at physical exposure characterization, is important, as it is rarely the exposure that matters, but rather the resulting therapeutic or adverse physiological effect. Even exposure safety guidelines, which are by their nature formulated in terms of physical exposure quantities (such as energy deposition or field strength) aim at preventing adverse physiological effects (such as thermal collapse or blood clot formation). Hence, there is value in directly modeling the ultimate, physiological quantity of interest, even if it comes at the cost of additional complexity and uncertainty. Providing physiologically functionalized anatomical models can reduce that effort and increase access to such modeling.

**Figure 4:**
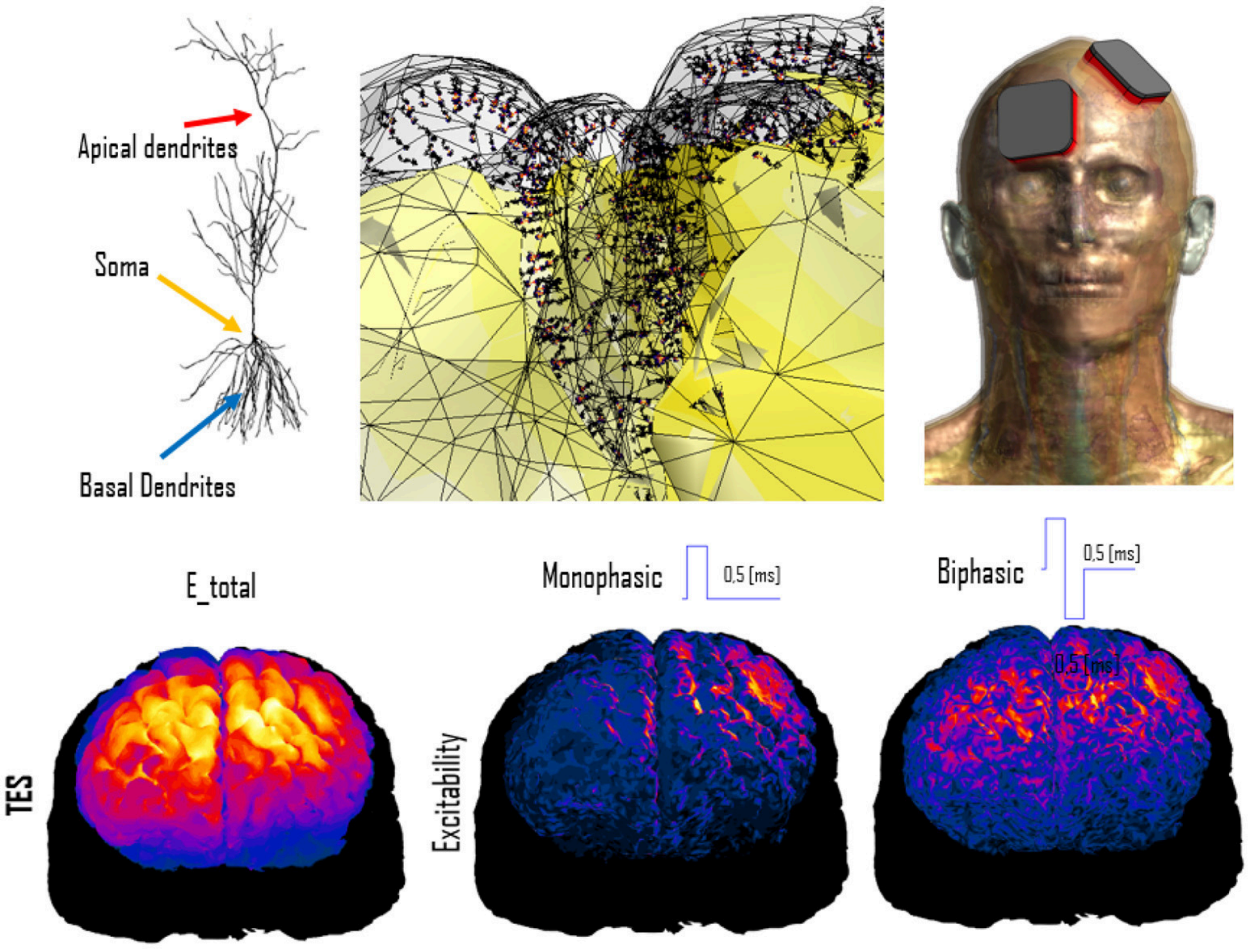
Simulation of transcranial electric stimulation. **(Top)** A detailed anatomical head model (right) has been functionalized with dynamic electrophysiological layer V pyramidal neurons (left) by integrating them at the correct anatomical depth within the folded cortical structure (center; the neuron coloring represents transmembrane potential). **(Bottom)** When exposed to modeled electric fields (left), the functionalized model can be used to computed neural excitability maps (shown for monophasic (center) and biphasic (right) pulse-shapes).

For example, functionalization of anatomical models with nerve trajectories (Figure 1) and with neuron electrophysiology models has proven valuable in questioning the assumptions behind current low-frequency exposure safety standards (Neufeld et al., 2016a,b; Reilly and Hirata, 2016) and gaining insights into relevant factors. Only by combining anatomical models that provide the physical environment in which exposure occurs with realistic neuron trajectories and electrophysiologically accurate neuron models it could be shown that (i) the inhomogeneous *in vivo* field conditions connect with the non-linearity of neural response to elicit action potentials and (ii) the discontinuity of these fields at tissue interfaces can give rise to neurostimulation in passing nerves. These forms of stimulation together produce safety concerns related to field inhomogeneity at relevant exposure strengths, which contradicts the mechanistic view underlying current safety standards. Current standards and safety limits are solely based on stimulation of nerve endings, which depends on field strength rather than field inhomogeneity. A new research agenda aimed at establishing the basis for revision of safety guidelines has been formulated to clarify these concerns (Reilly and Hirata, 2016). The agenda also includes a call for the development of new induction and electrostimulation models to update human exposure limits. Another illustration of neurofunctionalized anatomical models is the work presented in Cassara et al. (2017a), where a large number of electrophysiologically and morphologically detailed layer V pyramidal neuron models has been placed at anatomically realistic locations within a high resolution head model to investigate different forms of transcranial electric and magnetic stimulation (Figure 4). While the normal component of the electric field at the brain surface was found to correlate well with subthreshold neuron polarization (believed to be relevant for transcranial electric stimulation therapies), suprathreshold spiking excitability maps (relevant for transcranial magnetic stimulation) were found to poorly correlate with field exposure metrics. This demonstrates the value of using functionalized anatomical models to study the physiological impact, rather than stopping at physical exposure computation.

Functionalizing an anatomical model with a physiological network model of transient blood flow in major vessels (either 4D flow fields or reduced order models such as presented in Reymond et al., 2009) could support a wide range of modeling types: For example, it could provide realistic in- and out-flow boundary conditions for fluid-dynamics studies in anatomical vessel segments, or be used to simulate the thermal impact of discrete blood vessels during hyperthermic oncology therapies, or for pharmacokinetic modeling.

#### 2.2.4. Simulation and measurement results functionalization

An extension of the concept of anatomical model functionalization with image data is the functionalization with (physics) simulation results. There are many instances where simulation results are a generic first step to a more specific physics or physiology modeling step. In such cases, it can be valuable to precompute these results and provide them as a functionalization layer along with the individual anatomical models.

**Figure 5:**
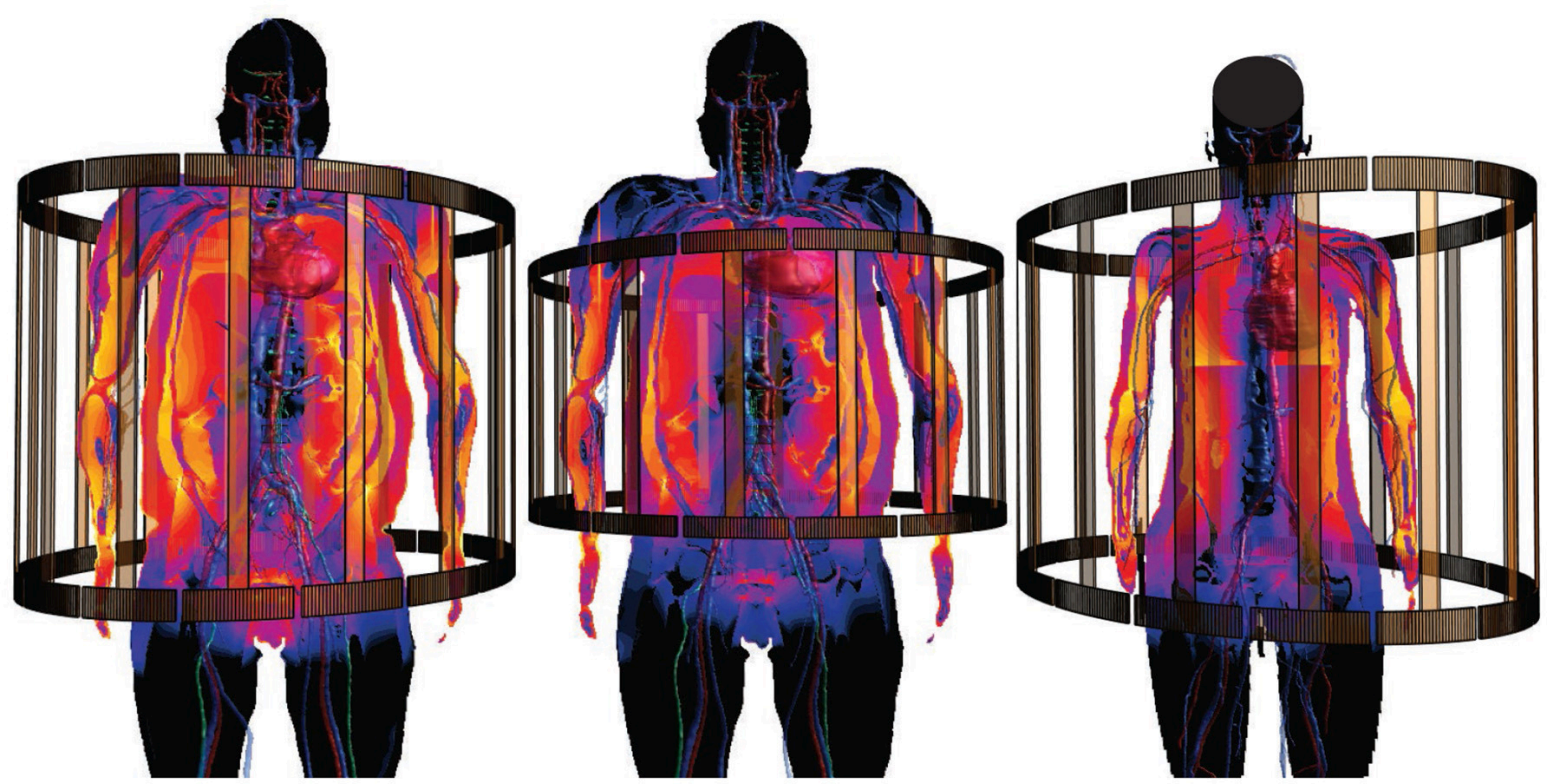
Anatomical models functionalized with incident *in vivo* field conditions from MRI RF-coil exposure, for use in MRI implant safety assessment (s. section 2.2.4). Different coils and anatomical models are shown.

For example, computationally determined EM exposure conditions (e.g., from far-field exposure) are frequently fed into subsequent modeling of, e.g., implant safety. This is the motivation behind the library of MRI RF coil exposure induced *in vivo* electric fields conditions (Cabot et al., 2016). Active implants exposed to MRI RF fields can pick up energy along the lead and deposit it at critical implant locations (e.g., the tip of a pacemaker or of an implantable neurostimulator), resulting in tissue heating and potential damage. According to ISO/TS 10974 standard (ISO, 2018), MRI implant safety can be ascertained by (i) determining the potential *in vivo* incident field conditions along lead trajectories, (ii) deriving the implant RF-heating characteristics (by measurement or simulation) as a transfer function from the tangential incident field to the resulting field/energy deposition at critical implant locations, and (iii) combining these two components to obtain the *in vivo* estimate of the energy deposition at critical locations. In a final step, (iv) the energy deposition is related to tissue heating. While the transfer function and potential implant trajectories are implant specific, the range of incident field conditions is determined by large permutations of coils, patient anatomies, and scanning positions. By precomputing a large set of *in vivo* field conditions, resulting from different coils, patient anatomies, and scanning positions, and storing them as functionalization layers of the anatomical models, it becomes possible to rapidly obtain statistical and worst-case information about a specific implant. One only has to identify possible trajectories in the anatomical models (which are used to extract incident field conditions from the field-functionalized anatomical models) and to provide a transfer function (Zastrow et al., 2016; Córcoles et al., 2017). This approach has given rise to some of the first successful regulatory submission based on *in silico* clinical trials (Brown et al., 2017). Libraries of field-functionalized anatomical models for 1.5 T MRI coils (Figure 5) have been made available to the ISO/TS 10974 (Annex P) for the derivation of conservative implant exposure.

Other examples of functionalization with simulation result layers include blood-flow velocity distributions or biomechanical motion displacement fields, which can be required information for implant effectivity assessment.

Similarly, it can make sense to store measured information as far as it can be localized or spatially associated with the anatomy in a functionalization layer. In addition to the use-case of providing input for simulations, measurement data can also serve the purpose of validation. For example, the ASME V&V40 standardization committee (ASME-V&V40-Subcommittee, 2016) employs the concept of validation evidence (Pathmanathan et al., 2017), which is the association of models with a collection of potential validation data, a selection of which can then be applicable to validate the model within a specific context of use. Illustrating examples of validation evidence functionalization include surface potential measurements (to validate electrocardiogram (ECG) modeling), flow velocimetry (to validate hemodynamics modeling), and compound action potential recordings (to validate neural dynamics modeling).

### 2.3. o^2^S^2^PARC

Based on the lessons learned during our extensive research over the last 15 years (see section 2.2), where specific functionalized anatomical models repeatedly offered the key to increased modeling realism, model reuse, and model potentiation, we put functionalized models at the core of our vision, design, and implementation of the o^2^S^2^PARC platform—the simulation platform and computational model integration center of the National Institutes of Health (NIH) SPARC initiative. Opting for functionalized anatomical models as integration centers offers a range of benefits:

- it integrates the computational models within their natural anatomical environment,
- it permits integration and coupling of initially disconnected, heterogeneous sub-models and advances interoperability,
- it permits the assessment of physical exposure by devices—the first step toward modulating physiology—which is frequently dependent on the local and sometimes large-scale anatomy with its tissue and physical properties distribution,
- it provides a framework to localize data and models according to their corresponding location within the body,
- it facilitates the identification of components for network or multi-scale computational models,
- it associates computational models with data, potentially originating from other users, that can be valuable to feed, tune, or validate the computational models.

The goal of this section is to provide a short description of the o^2^S^2^PARC platform as a specific example that highlights the value of centering unified computational frameworks or platforms on functionalized anatomical models.

**Figure 6:**
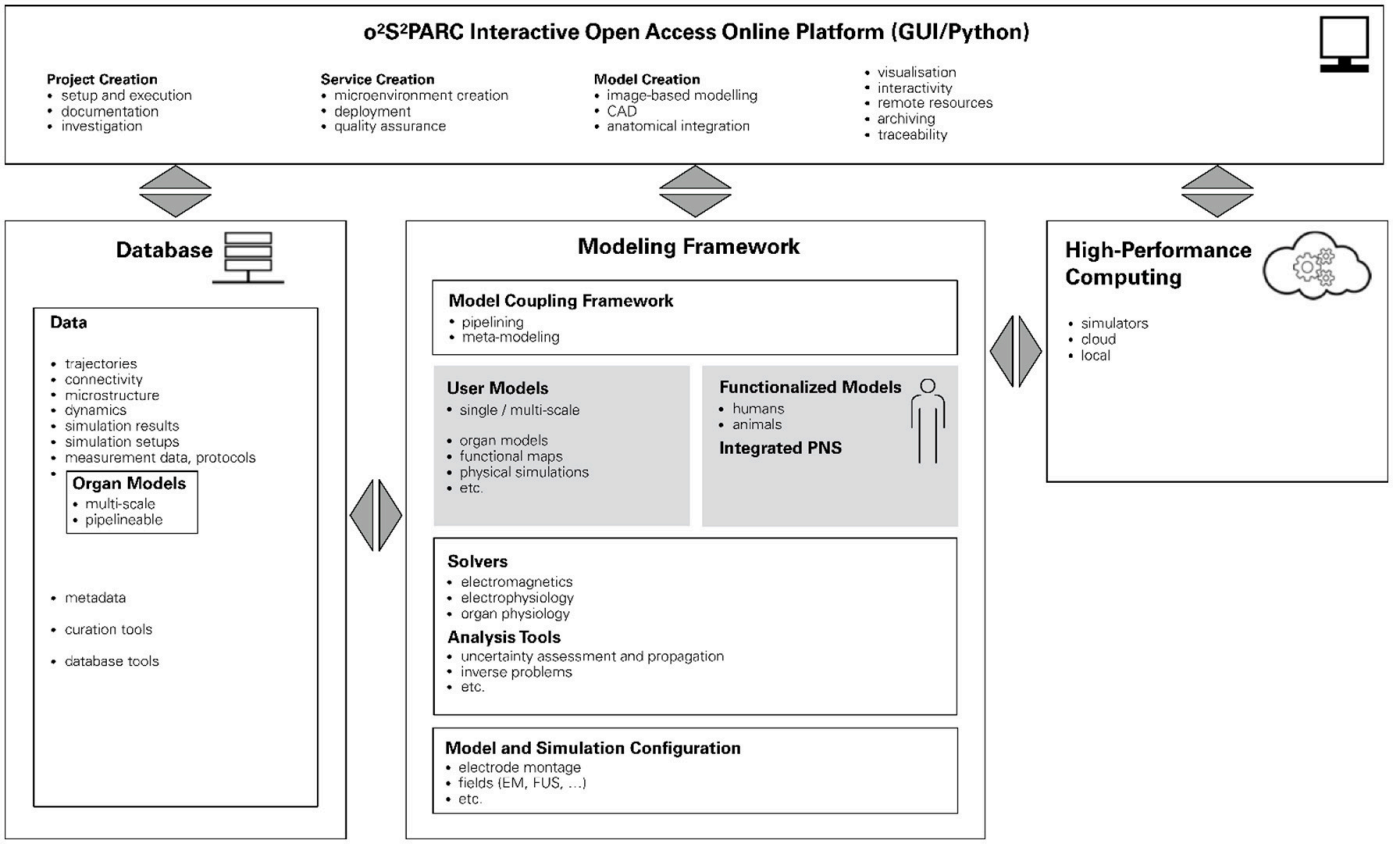
Schematic illustration of the o^2^S^2^PARC platform. At its center are functionalized human and animal anatomical models which serve as integrators for computational models (and data) from SPARC. The modeling framework is complemented by physics and physiology solvers and analysis tools. In combination with configuration information, studies can be composed that are then evaluated locally or in the cloud. All underlying data (including versions of the employed solvers and computational models) are routed through a database to ascertain full chain-of-custody and support quality assurance. The platform is accessible through a user-friendly online GUI.

The NIH SPARC initiative is an ambitious program to study the PNS and its role in controlling organ physiology toward the goal of being able to modulate the PNS to influence organ function and precisely treat diseases. In order to achieve sustainability and broad usage of the results of this research initiative, the SPARC data resource center (DRC) was established. One arm of the DRC supports the development, installation, and maintenance of a freely accessible online platform to host, run, couple, and study all computational models developed across the SPARC community. Our (funded) proposal for the design and implementation of this platform is based on our belief that this ambitious goal can be only achieved by the presented paradigm of functionalized models. At the center of the o^2^S^2^PARC platform will be the so-called “NEUROCOUPLE” and the “NEUROFAUNA” (Figure 6), detailed anatomical representations based on segmented image data (initially a male and female human model, as well as a rat model) that include an extensive tracing of PNS trajectories. These models are complemented by a more abstract body representation established by the SPARC MAP-CORE team, which is primarily used to represent dependencies, coupling, and multi-scale models, and to map information between models. The anatomical models will increasingly be functionalized with data and models elaborated within SPARC and, as such, serve as integration centers for the knowledge generated by that initiative.

**Figure 7:**
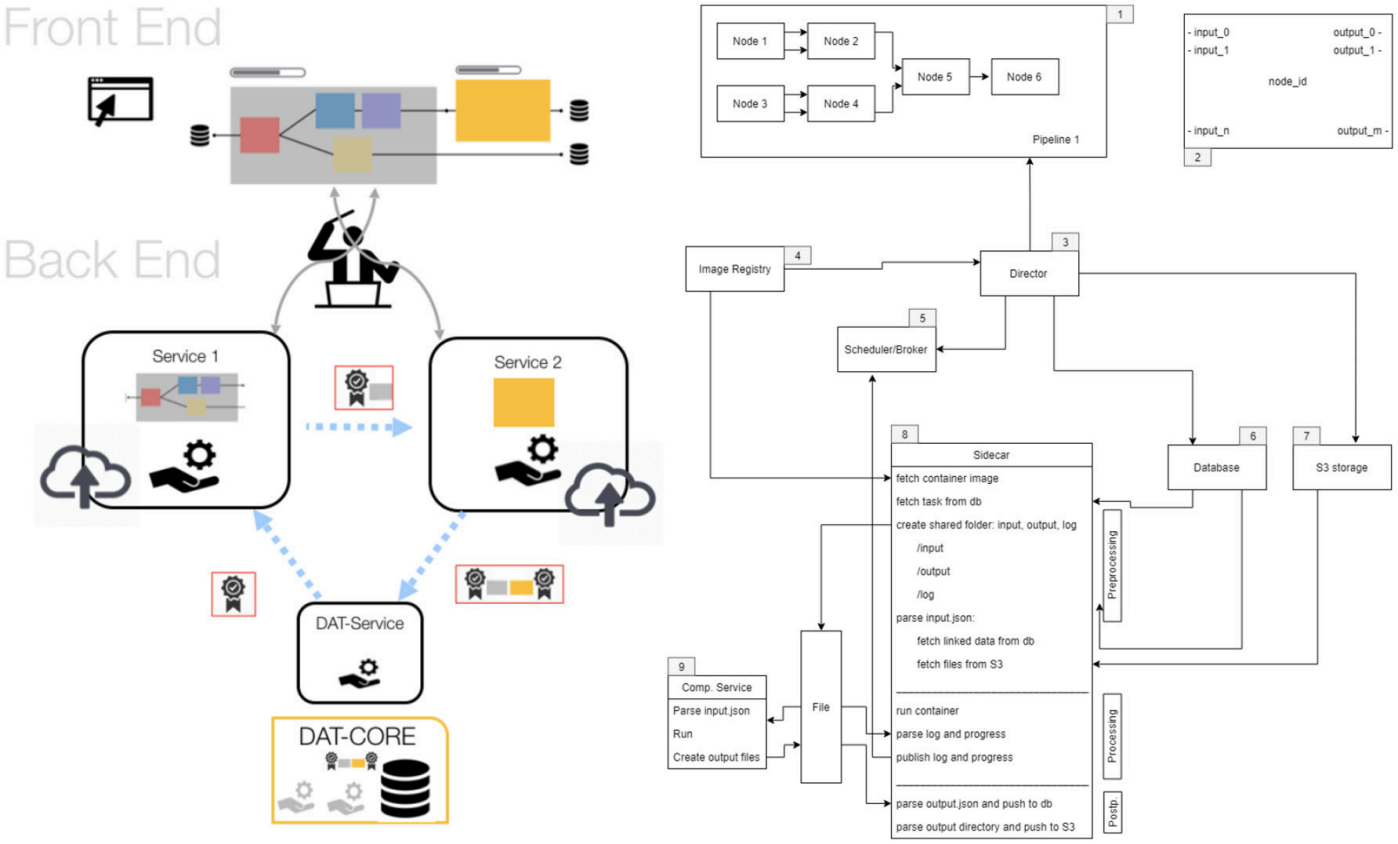
Structure of the o^2^S^2^PARC implementation. The online GUI communicates with a director that orchestrates a scalable network of computational service modules (left). The components of the backend: 1. computational pipeline, 2. nodes, 3. director, 4. Docker image registry, 5. scheduler/broker, 6. database, 7. S3 storage, 8. sidecar, and 9. computational services. For more information, see https://github.com/ITISFoundation/osparc-simcore.

Functionalization of the anatomical models in o^2^S^2^PARC started by including electrophysiological models of PNS neurodynamics. Further functionalization with anatomical microstructure, e.g., of the different nerve tissues (fascicles, epineurium, perineurium), statistical neuron distributions, and functional sub-units, will enable the investigation and design of different neuromodulation devices, by allowing modeling of the stimulator physics (e.g., EM or ultrasound exposure) and the induced electrophysiological response. Different SPARC teams are developing, often multi-scale, models of organ physiology, which typically accept PNS activity descriptors as input and output. Those physiological models will also be added as part of the model functionalization to simulate organ electrophysiology and the impact of PNS neuromodulation on organ function. o^2^S^2^PARC allows to execute computational models locally, or in the cloud, as microservices (see below), and to couple multiple such models, with a primarily electrophysiologically driven coupling concept—meaning that the data exchange between computational physiology models will typically be in terms of neural activity quantities (spiking frequency, transmembrane potential traces, network activity, etc.). Much of the SPARC activity currently focuses on establishing enervation maps, neural tracing, and connectivity information, which will be integrated with other data and models by mapping to the whole-body anatomy. Other data being collected within SPARC and valuable for model functionalization includes image data (e.g., enabling the creation of high-resolution sub-region anatomical models), organ motion data (e.g., to phenomenologically describe activity of the gastric system), and electrophysiological measurements (e.g., large-scale neural activity recording, sometimes synchronized with related organ physiology recording).

o^2^S^2^PARC is not limited to the integration of data and models within functionalized reference anatomical models, the creation and execution of computational models, and their coupling, but also foresees a range of meta-modeling (e.g., optimization, inverse problem solving, uncertainty assessment), versioning (e.g., derivation of updated/modified models), and quality assurance (e.g., reproducibility, verification and validation, quality certification, chain-of-custody, referencability) functionalities. o^2^S^2^PARC will also offer a range of physics simulators (e.g., EM solvers).

o^2^S^2^PARC is an open-source (MIT License) project hosted at https://github.com/ITISFoundation/osparc-simcore, (see also for further technical information). The o^2^S^2^PARC platform is realized (Figure 7) as a web-browser-based graphical user interface (GUI) front-end implemented in Qooxdoo that communicates through RESTful and web-socket APIs with the Python-based web-server back-end. The web-server, in turn, communicates with a Python-based director that orchestrates a scalable network of computational service modules. Inter-service communication between computational services is restricted to file sharing. Each service module is encapsulated within a Docker microservice (a concept similar to virtual machines, but with less computational overhead, that allows to execute programs within flexibly configurable environments) and paired with a “sidecar” that is responsible for the monitoring and command of the associated computational service (including communication with the director). Encapsulating the computational services within Docker containers ensures that (i) the services can run anywhere (locally, in the cloud, or on an in-house cluster) within a known and tested environment (libraries, operating system, compiler, etc.), (ii) the services and their environment can be archived together in a docker repository to ensure future reproducibility, and (iii) services can be implemented using a wealth of available docker environments (scripting languages, Octave, command-line executables, C/Java compilation and execution, Jupyter Notebooks, machine learning frameworks, etc.) while maintaining a standardized communication interface and protocol through the sidecar. This ensures that service providers can implement their computational models and services flexibly, using familiar technologies and environments, and do not need to convert them to imposed technologies and forms. Services are typically setup as pipelines (or more generally as graphs) which can include, e.g., geometric or image-based model creation, data-sources, physics and physiology solvers, visualization and analysis modules. The functionalized anatomical models themselves will be provided as service module, providing visualization and searchability (e.g., through ontologies and knowledge-graphs elaborated by the SPARC MAP-CORE team), and offering geometry and functionalization layers as selectable outputs that are then further processed/employed throughout a project's graph.

## 3. Discussion

The results obtained using functionalized anatomical models presented in section 2.2 illustrate the value and importance of bringing anatomical representation, information supporting modeling and model validation, as well as physiological dynamics closer together and synchronizing them. Combining these factors not only helps to ensure consistency, but is also a way of leveraging the value of individual contributions to modeling and of integrating parts into a bigger total. Once validated, functionalization layers can become established model components for third party users, just like anatomical models currently are. An important benefit of the functionalized anatomical model paradigm is that it can also facilitate interoperability for heterogeneous models developed by research group from different modeling backgrounds and fields.

The functionalized anatomical model concept presented here does not stand alone. It is related to other initiatives, such as the IUPS and VPH Physiome Projects mentioned before (see also section 3.1) and—when coupled with model personalization—to the concept of the digital patient ‘avatar’ and precision medicine in general (section 3.3).

### 3.1. Physiome projects and the functionalized anatomical models concept

The Physiome Project was initiated by the IUPS in 1997 to bring multi-scale engineering modeling approaches to the physiological interpretation of the vast data, from the molecular to the organism level, that was becoming increasingly available (Hunter and Borg, 2003; Hunter, 2016). It aims at providing a quantitative, multi-scale description of physiological dynamics and functional behavior of the intact organism. The Physiome idea has produced a range of initiatives, such as the VPH Physiome Project, which emerged from the European Commission VPH Project, in combination with the US Interagency Modeling and Analysis Group (IMAG) initiative and others (for more background on different Physiome Projects and related initiatives, see http://tutorial-on-cellml-opencor-and-pmr.readthedocs.io/en/latest/background.html). Particularly the VPH Physiome Project pursues a vision where models and sub-models are reproducible, reusable, and discoverable (Nickerson and Hunter, 2017), e.g., by elaborating standardized markup-language descriptions of computational models, data, and measurement protocols. The project aims to describe the Physiome through ordinary and partial differential equations (ODEs and PDEs) that are made compatible, e.g., by enforcing energy and mass fluxes through Bond-graph theory (Paynter, 1961; Safaei et al., 2018), and by employing standardized naming conventions/ontologies and formats (e.g., CellML, SBML Chaouiya et al., 2013, FieldML) that are in turn compatible with general purpose solvers such as OpenCOR and OpenCMISS (Hunter, 2016).

The functionalized anatomical model concept is, in parts, a realization of the Physiome vision in that it also pursues the integration of computational physiology models, with the goal of achieving model reuse and potentiation, as well as a more comprehensive description of the organism. However, it is also distinct on several levels:

- The functionalized anatomical model concept emerged from the need to consider the human anatomy and body as an environment that is subject to physical exposure or within which physical exposure occurs that interacts with physiological activity. The interaction with physiology can be intended (e.g., therapeutic) or unintended, in which case human safety is the motivation. Hence, the anatomical geometry, the tissue and external environments, and the modeling of physical exposure and physical processes in and around the body are central. In contrast, the Physiome Projects have a holistic approach: they aim to explain how each and every component in the body works as part of the integrated whole by establishing a multi-scale model of the physiology.
- The multi-scale physiology models in Physiome Projects can in some cases be related to the spatial distribution of molecules, cells, tissues etc. and therefore can also involve anatomy. However, anatomy is only a potential contributor to the computational physiology model, while the interconnection between anatomy, physical interaction, and physiological response or dynamics is at the center of the functionalized anatomical model concept. Whereas conservation of mass and energy fluxes serves as primary integration frame in the VPH Physiome Project (Hunter and Viceconti, 2009), it is the anatomical space that is central in functionalized anatomical models. The anatomical space not only allows one to integrate sub-models, but also serves as a coordinate system for locating and storing other information, such as measurement data.
- The Physiome vision (Hunter and Borg, 2003) pursues a strong integration of sub-models through standardized (markup language) formats, descriptions, and meta-data. Its ultimate goal is to accumulate mathematical descriptions of physiome components at all model scales resulting in an emerging organism physiome model that can be solved using dedicated software such as OpenCMISS and OpenCOR. It employs methods, such as Bond-graph theory, to ensure flux conservation and physico-chemical consistency.
  While we embrace these initiatives for unified descriptive or markup languages and model databases, wide adoption of such markup languages is still lacking. The process of a posteriori converting established models into Physiome Project-compatible models and descriptions is often a considerable burden for groups. In addition, models are often implemented using numerous custom or proprietary software tools, which do not (yet) support Physiome standards and formats, and which are difficult to replace with other tools for performance or robustness reasons. Furthermore, not all forms of modeling are amenable to mathematical descriptions that can be used to ensure flux conservation—for example, machine learning produces models that frequently have no known mathematical description or guaranteed conservation. We believe that a more heterogeneous and flexible integration approach is needed to ensure a wide adoption and the emergence of a broad range of functionalized anatomical models, even though this comes with the expense of typically being limited to looser integration and weaker coupling.
  Recent progress—in particular the emergence of Docker microservices, with the wealth of software tools becoming available in dockerized form and the simplicity of creating and deploying such services—enables the easy integration of heterogeneous models developed with widely differing technologies, as proposed in the o^2^S^2^PARC platform. The platform is designed to facilitate orchestration and communication between the services and only requires to provide translators between outputs of one service and inputs of another service.

### 3.2. o^2^S^2^PARC and beyond

Realizing o^2^S^2^PARC permits to demonstrate the value of the functionalized anatomical model vision, as a means of reflecting the tight interconnectedness between anatomical geometry, physics, and physiology, and of providing users with leveraged benefits from data and computational models developed by others as stepping-stones and building blocks for their own models and studies. The open-source, online technology generated for o^2^S^2^PARC is sufficiently general to be of broad usability for a large variety of computational life sciences communities and applications.

To take this forward, the CLS community needs to agree, at least at the level of application-specific communities, on standardizing at the minimum (i) coordinate mapping between anatomical models and functionalization layers, (ii) descriptors of required input and provided output for computational models, to facilitate model coupling, as well as (iii) data descriptors, minimal data standards, and ontologies. Some standards and ontologies have already been developed, e.g., in the VPH Physiome Project, and remaining close to these standards will maximize compatibility. Additional valuable meta-data include information about the degree of verification, validation, and certification, corresponding experimental data, unique identifiers (also to facilitate referenceability), documentation (for example: How was a model/data generated? What are the associated uncertainties? How is it correctly used?), version log, related publications, usage history, access- and copyrights, to name a few. As already mentioned, the right balance between facilitating compatibility and avoiding high integration are significant challenges for model and sub-model creators. Means to incentivise this additional effort must be found. Apart from offering model integrators the benefit of increasing the use and usefulness of their own models and giving them access to third party integrated models, platforms such as o^2^S^2^PARC serve as powerful integration motivators, as they afford model creators with a complete platform infrastructure that enhances the model—with minimal effort—with features such as an attractive GUI and support for cloud execution.

### 3.3. Personalized functionalized anatomical models

Another level in the value of functionalized anatomical models can be achieved through model personalization, which supports and enables a wide range of additional applications, e.g., in precision medicine or *in silico* clinical trials.

As repeatedly illustrated in section 2.2, image-data offers a way to personalize functionalized anatomical models. At the scale of the whole body, organs, and tissues, personalization is achieved by registration of a reference anatomical model to patient- or subject-specific image-data. For functionalized anatomical models, the functionalization layers can be transformed along with the anatomy. Once the anatomy is co-registered to the subject-specific image-data, further-going use of subject-specific image-data can be made, to personalize tissue property distributions, boundary conditions, organ motion, and more.

Personalized functionalized anatomical models can be valuable for personalized safety assessment (e.g., dosimetric evaluation of MRI exposure based on patient-specific anatomical geometries to relax restrictions of scan sequences Murbach et al., 2017b) or for patient-specific treatment planning (e.g., personalization of hyperthermic oncology treatments through optimization of the energy deposition considering thermoregulatory response Paulides et al., 2013). The latter can be seen as an implementation of the “Precision Medicine” concept, where healthcare is customized and tailored to the individual patient through patient-specific data obtained, e.g., from molecular diagnostics, imaging, and analytics. An extension of the use of patient-specific anatomical models is the concept of digital patient “avatars” (Maniadi et al., 2013; Brown, 2015), “a vision for the digital representation of personal health status in body centric views. It is designed as an integrated facility that allows collection of, access to, and sharing to life-long and consistent data.” (Maniadi et al., 2013). The digital avatars concept envisions comprehensive digital models which allow to integrate patient-specific information, such as diagnostic measurements and computational models, in order to provide a comprehensive and accessible patient picture, tailored predictions of disease progression and therapy outcome, and to facilitate precision medicine. Large scale, ideally automatized, creation of personalized functionalized anatomical models will support the digital avatar vision and will also result in patient functionalized anatomical model populations. Such functionalized anatomical model populations are valuable for the assessment of variability, e.g., of dosimetric exposure or therapeutic impact. They are fundamental for the realization of *in silico* clinical trials, where sufficient coverage of the target population is required.

## 4. Conclusions

In the last 20 years, the use of computational anatomical models has driven the field of CLS to new heights and as such they have become an indispensable staple. More recently, the possibility of model functionalization with geometry parametrization, physical and physiological dynamics, and simulated/measured data has empowered researchers to take another major step forward (i) by increasing model dynamism and realism through consideration of the coupled nature of anatomy and physiology, and (ii) by supporting reuse of established models/data. The development of functionalized models and the related techniques has already started to broaden the application range of CLS dramatically, e.g., in *in silico* treatment optimization and personalization, medical device research and development, as well as safety and efficacy assessment. In the near future, it is expected that this approach will bring CLS even closer to the goal of replacing clinical human trials with *in silico* human trials.

Functionalization properly accounts for the interconnection between anatomical geometry and bodily dynamics—both the impact of physiology and motion on the anatomical geometry and the impact of changes in anatomical geometry on the environment within which physical exposure occurs, which in turn affects physiology. It leverages the value of individual model components, especially if care has been taken to ensure consistency between the underlying anatomical model and the functionalization layers. Functionalized anatomical models can serve as natural integration approach in collaborative efforts, as illustrated by the introduction of the paradigm for the o^2^S^2^PARC platform. Furthermore, functionalization with measurement information can help realize the vision of validation evidence, as formulated, e.g., by the ASME V&V 40 committee, toward scientifically sound validation. The value of functionalized anatomical models has already been demonstrated in a wide range of application, but it is a very general concept, suitable as one of the fundamental components of CLS.

The integration of models and sub-models within functionalized anatomical models can be both facilitated and advanced by reducing the model integration effort through the support of heterogeneous model functionalization—one of the main aspects of the o^2^S^2^PARC platform—and by offering the motivation of benefiting the model with the functionality of a comprehensive computational platform, including an attractive GUI and cloud computing.

## Author contributions

All authors contributed to the formulation of the functionalized anatomical model vision and provided input affecting all of the paper content. The first manuscript version has been drafted by EN. The manuscript has been extensively reviewed and revised by NK. WK has been fundamental in driving the development of the Virtual Population and in first formulating the vision of o^2^S^2^PARC. BL has contributed particularly to sections related to model shape parametrization (morphing, position, etc.), and to specific anatomical models. BS helped with literature review and interpretations of the contribution of the paper and revised the manuscript accordingly.

### Conflict of interest statement

The authors declare that the research was conducted in the absence of any commercial or financial relationships that could be construed as a potential conflict of interest.
